# Opening Address, Delivered before the American Society of Dental Surgeons at the Annual Meeting Held in Philadelphia, August, 1851

**Published:** 1852-07

**Authors:** J. H. Foster


### ARTICLE II.

*Opening Address, delivered before the American Society of Dental Surgeons at the Annual Meeting held in Philadelphia, August,* 1851. By J. H. Foster, M. D.

"We are all grave, serious men, in our way; we think it our duty, as far as in us lies, to take care that the constitution receives no harm. To censure doctrines or facts, persons or things, which we do not like. If other people are not of our opinion we cannot help that. It were better they were."

Addison.

Mr. President and Gentlemen *of the American Society of Dental Surgeons*:—May I express the belief, that we have met here to-day, on this our 12th anniversary, with the ardent desire, one and all, to labor zealously for the community, strenuously for our friends, and sufficiently for ourselves. Let us endeavor so to apply ourselves, that, with God's blessing, we may have reason to felicitate each other, when our labors are closed, that our assembling together shall have been productive of more valuable information and matter conducive to the public weal, than we have realised from the past.

This is the second time you have entrusted to me the honorable and no less pleasurable task of presenting the Annual Address.

Would that I might render, in the performance of that duty, some adequate return, not an equivalent, by any means, but some compensation for the spirit of mutual confidence and trust, which, animating one side, ought, at the same time, to actuate the other to fulfil his obligations.

May I indulge the hope that the same feelings which induced you to bestow upon me that renewed flattering testimonial of your esteem, will induce you also to extend that charity and indulgence which a keen sense of my own insufficiency impels me to solicit.

Eloquent voices, inspired by the emotions which occasions like these are calculated to produce, might, from the fire of their own souls, enkindle in yours, that spirit of emulation, of zeal, of united and persevering effort, "in every good word and work," tending to increase and magnify the honor and usefulness of this body.

Distinguished members—there are among you, who might give utterance to words of wisdom, and by their reason and good sense, in earnest appeals to your judgment, succeed in determining you to new means and new measures, to insure peace, harmony, and happiness in our councils, and promote the best good of the profession.

Sir, it is impossible for us to be blind to the fact, that there have been periods of great anxiety, peril, and danger, in the history of this society; the causes have been too often reverted to, and are too well known to every member, who had any part or share in its establishment, to require their recapitulation. If we were to trace them to the original source, we should discover that there was one primary cause of all our difficulties and distractions, and we should concur in the opinion, that all the violence and intemperance, which have characterised its career, has been owing to the growth of party spirit, emanating from the vain attempt, to establish a society upon the broad basis of a liberal and unrestricted extension of universal fraternity.

It is not my intention, however, to enter into a discussion of these topics.

In regard to these, my opinions and views have been freely and sufficiently expressed. We were compelled to adopt the motto, "divide and conquer," to save ourselves from disgrace and defeat, nor do I believe that any thing else could have been done, any other course taken, by any or all the consistent members of this association, to avert the effect which such causes must of necessity have produced.

There is no longer the material or the question, out of which can emanate that spirit and intemperance of party, nor that determined and resolute advocacy of a particular system of malpractice and imposition, to be carried on by a minority of the members, under cover of the sanction, and in the name of this association, which the past has witnessed.

There is no longer the evil spirit of contending faction, led on by the black demon amalgam, to throw the fire-brands of discord and disunion into our ranks, but there may be, gentlemen, questions, points of order, other issues at stake, in your future deliberations, as there have been in the past, that will require all that discrimination, which, as thinking, reflecting, enlightened men, you can bring to aid you in the performance of your public professional duty, and in maintaining the high position for this society, which it was expected and designed to occupy.

It would be vain to arrogate to ourselves entire immunity from error—"to err is human," and from incidental causes, the action of association, as well as of the individual, may be affected in drawing conclusions from unfair or mistaken inferences, and though the verdict may be rendered in strict accordance with the evidence presented for our judgment, through a distorted view of facts, we may err in that judgment.

While, therefore, we are inclined to lean to the side of mercy, and are, perhaps, prone to exhibit leniency, when individual character and professional reputation are to be considered; let us be cautious to do no injustice to ourselves, and thus leave the profession and the community without those safeguards, which it was one object of this association to provide.

On the other hand let us be equally careful, in deciding weighty questions, that while we are firmly resolved to make no sacrifice of the eternal principles of "truth and justice;" but "to mete to every man his measure," that in relation to all matters involving uncertainty and doubt, and in which the evidence is not full, entire and satisfactory, the scale shall preponderate on the side of mercy.

Mr. President, what vicissitudes have we not experienced in the short period of our corporate existence. Twelve annual revolutions of the sun mark the age of this association; and, sir, I ask what have we accomplished, compared with what we might have done, to advance the growth of science—to cultivate and improve our art, and enlarge our sphere of usefulness? We cannot shut our eyes to the truth, that in times past our best efforts have been counteracted by the adverse winds of faction. We must know and we do know, that the party spirit, to which I have alluded, has prevailed more or less, in all our deliberations. That heat and passion, engendered by the existence of this spirit, has mingled in our councils, and menaced the existence of this society, even after we thought we had exorcised it, and instead of uniting as one man to extinguish the flames, we seem to have been eager in searching for new fuel to feed this corroding fire, and in inventing compound blow-pipes, to concentrate this devouring, blasting, unsatiated element.

I come here, to-day, unable to utter one word upon any subject, other than this, for my thoughts would dwell upon no other, until the spirit of peace and fraternity had been invoked, to guide us in our future councils.

I implore all members who have come here this day, I entreat them by all that is dear to them in connection with this society, by all that they hope of good to be derived from it, and of good to be accomplished by it hereafter, to do all in their power to repress these passions, to look to its interests, to listen to the voice of reason, not as it shall be uttered by me, for I am not vain enough to indulge the expectation, that any thing I can say, will avert those evils; but to listen to their own reason—their own good sense—their own judgment in determining what is best to be done in the future, to accomplish its ends and objects.

Let us, as far as practicable, endeavor to accommodate and reconcile all questions of differences, upon which we may be unhappily divided, with a conviction of the intimate connection which exists between the individual members, who compose this body, and the body itself, and with a deep sense of the utter impossibility, that the body can ever accomplish any great or useful end, so long as its members continue to be at war one with another.

All adjustments, and all arrangements of controverted questions, may generally be successful and effectual, if both parties will make some concession, not of *principle* by any means—not of principle at all; (to use the language of our greatest statesmen,) "we stood firmly upon that platform once, and I trust we shall always stand there," but some concession of feeling and opinion, upon matters which do not involve any sacrifice of principle, may be sometimes made without individual loss or disparagement, for the "greatest good of the greatest number."

I do not intend, sir, to argue this or any other question at this time. I came here to express only opinions and impressions, and leave it to those who hear them, to debate the matter in their own minds, and draw their own conclusions.

We all know the great prominent substantial object for which this society was founded, and to the fulfilment of this purpose, let us direct our energies: let it be the leading and controlling idea: let us sink all secondary inferior considerations, to this primary, most important object. Looking anxiously at the true condition of things, and the nature, purposes, and ends for which we are convened and organized, let us act "*bona fide ad id finis*," in the fulfilment of all those obligations, which, as men of honor, we are pledged to hold sacred and binding, in supporting the constitution and laws, to which we have subscribed.

What are the subjects which most demand our serious consideration? How shall we best subserve the interests, and supply the wants of the profession and the community?

What measures shall we take to extend the means, for the largest increase of useful knowledge?

These and many other questions might be asked, which would consume all your time for many years, to think and act upon, and the answers to which, in the fund of information afforded, by the combined power of all your minds, might fill volumes, for those who are now looking to you for new light and revelation, establishing a sure foundation for those who come after you, upon which to build a superstructure, that shall redound to your honor and immortality.

There is nothing which so effectually represses all interest in, and desire of, associating with, this society, as the disposition evinced to waste time upon matters foreign to those which should engross our attention.

We meet together once every year for mutual improvement. The professed and avowed object is, to increase the general stock of knowledge, yet, a great portion of the time is passed in useless discussions; neglecting to avail ourselves of the light, now dawning everywhere, upon this great science, (for it is a science as much as astronomy, geology, or anything else,) we go away from our meetings with very little benefit, or improvement, compared with what might be obtained, if our proceedings were well ordered. Nothing in the world could do so much for the promotion of this great interest, as a *system* of education, aiming at a knowledge of the wants of the profession, and the means of supplying them, so that the material may be formed, to answer the increasing demand for good operators.

There are many who pursue the same course of practice, that others did forty years ago, and are content to do so. They do not aspire to any more knowledge than they already possess. "Where ignorance is bliss, it is folly to be wise."

Our duty is, to assist those who are seeking for information, and who will be benefited, if rightly instructed. Those who are impressed with an earnest conviction, that there is no goal in the acquisition of knowledge, that the field of scientific research is illimitable, boundless as the universe.

Let us do all in our power to strengthen those impressions, to fix indelibly these convictions upon the minds of those who are entering, or who have already made some progress, in the path of professional attainment.

Let them be made to understand, that the world is getting too practical to help drones and push them along, when there is a busy hive of workers.

The mind has a certain vegetative power which cannot be wholly idle. If it is not cultivated into a beautiful garden, it will, of itself, shoot up in weeds and flowers, of a wild and noxious growth. This idea suggests to my mind the importance of paying more attention to DENTAL LITERATURE.

We are not a reading profession. Our students accomplish all that is required to secure their diplomas, and too often afterwards become mere *mechanical puppets.*

The string has been pulled, and the hands and fingers set in motion. They may perform their work well. But is this all there is to do ? This all that is expected of him ? Is it for this, and this only, they have read and studied ? Is the pathological and physiological knowledge, which they have acquired, in your schools and colleges, to exert only a temporary influence upon their professional character ? Have they not been taught, that this knowledge sustains an intimate and important relation in the sympathetic connection which exists between other organs in the human frame, and those to which their attention is more especially to be directed ? Have they not genius and capacity ? Have they not a laudable ambition to bring these faculties into active exercise ? To extend their researches—expand their intellects—beautify and adorn their characters, not alone by exclusive devotion to dental literature, but by a general and select course of reading, which shall render them in scientific acquirement, in ability to impart pleasant and satisfactory information upon a variety of topics, ornaments to society, as well as to the profession ? We need more *educated* men ! We need them in *society*, for, ever since *literary* men have entered upon this field of labor, the distinction has been in *their* favor, and they have assumed the higher social position, which learning, combined with practical skill, entitles them to occupy. We need them in the *profession* to give greater *character* and *influence* to its increasing importance. We need them as *editors* to some of our public journals, as well as *contributors.*

This profession claims the strictest alliance with the cause of humanity, it proffers substantial blessings to man. It has an extensive scope ; it investigates the structure of that beautiful mechanism, which God has given us for a most useful purpose. It searches and examines into the laws of its vital movements, both in health and disease, and their sympathetic connection with the whole animal economy. It contemplated a great variety of influences, by which its complicated processes of development, (too generally prone to a morbid character and action,) are accelerated, retarded, suspended, or destroyed, and therefore to shield society from the pain and suffering, which ignorance, or custom, or recklessness, may entail upon it, do we especially need *educated men.*

Let, then, our societies, colleges, and private schools of instruction, be impressed with the weighty responsibility resting upon them, with the due importance of inculcating a more continued and unwearied application in the pursuit of knowledge, upon the part of those who are just entering upon their probation.

Students must labor long and earnestly before they can realise how much there is to be accomplished in obtaining all that is intrinsically important to fit them for practice, and that in a whole life time of application to this end, they will have gathered comparatively little of the accumulated amount of useful, revealed, as well as yet undiscovered treasures, which are to be extracted from the vast store house of knowledge, the doors of which are always open to the votaries of true science.

Let them study all the duties they owe, in the largest sense, to the communities with whom they live and labor.

Let them be able to explain the mechanism of the physical organization of the teeth, and more than this, to expound, so far as known, the beautiful and harmonious laws enstamped upon the whole organization of the human frame, by which its complicated movements, and diversified phenomena are sustained. Laws as immutable in their nature, and inflexible in their operations, as those which hold the planetary system in their order. Let them in literary attainment, in scientific research, fit themselves to instruct, and even should they never be called to fill public places as teachers, they will find opportunities in every day life, to avail themselves of their advantages, to their own advancement and usefulness, and the good and happiness of all around them. No man I believe was ever yet conscious of possessing too much knowledge of a useful character. How many have to mourn over their deficiency in this particular, and how many, alas, deeply sensible, that through their own neglect they have lost the opportunities, which might have fitted them to occupy stations of greater honor, confidence and trust.

"Richard Burke being found in a reverie, shortly after an extraordinary display of powers in the House of Commons, by his brother Edmund, and questioned as to the cause, replied, I have been wondering how Ned has contrived to monopolize all the talents of the family; but then, again, I remember when we were at *play*, he was always at *work*. The force of this anecdote, is increased by the fact, that Richard Burke was considered not inferior in natural talents to his brother: yet the one rose to greatness, while the other died comparatively obscure." Trust not to your genius, young man, if you would rise, but *work! work! work!*

Let your pupils comprehend, too, that they are not to be servile imitators of any man's operations, that they themselves have inventive faculties, and that those faculties must be cultivated, that they, too, may furnish their quota, to the great treasury, into which so many in this "golden age," are pouring almost exhaustless contributions of individual enterprise and skill.

My greatest fear, I must confess, is, that the rules and requirements for the admission of candidates into our schools and colleges, are not sufficiently exacting, or not rigidly enforced; that too often students have been matriculated, without having received such a preparatory education, as would enable them rightly to improve the advantages, which they expected to derive from a course of instruction.

Notwithstanding our rapid advance in theoretical and practical knowledge, it appears to me, as if our profession, as its numbers increase, is becoming in a greater ratio, more and more mechanical.

To what is this to be attributed? To the great improvements which have taken place in the manufacture of mineral teeth, and in the adaptation of plates. Many operators are too ready to condemn *natural teeth,* which might be made useful and serviceable for many years, because it is easier to insert artificial ones, or because they are unwilling, or *unable* to perform those elaborate and intricate operations, which are essential to their preservation. The operation of the excision of the nerve, and the filling of teeth into the fangs, might be performed successfully in thousands of cases every year, in which, through ignorance, or culpable advice, such teeth are sacrificed, and the patient is induced to believe that a mineral appendage, is a more ornamental, if not useful substitute.

Let those who show a particular devotedness to, and the greatest proficiency in, the operation of *filling teeth,* be impressed above all with the conviction, that those who are attaining pre-eminent rank and reputation at the present day, are those who are performing new and untried operations.

Let them attempt, and persevere in their efforts by repeated trials and experiments, to effect success in filling the largest and most difficult cavities with *gold,* even in cases in which success is almost considered hopeless.

Let them be encouraged by the reflection, that one operation of such a character, one cavity filled which contains ten or twelve sheets of *gold foil, one tooth saved,* under such circumstances, by a patient and laborious expenditure of time and talent, *will,* though it may not manifest itself for many years, *eventually* do more, if perseveringly continued in, to establish a reputation for superior practical skill, than the *ninety and nine* ordinary operations in the hundred.

But to the accomplishment of this end, there must be an entire conviction of the truth of this proposition.

There must be a will strong enough to overcome, and capable of subduing any and every opposition of the mind, and compelling its assent. There must be no "halting between two opinions."—There must be no doubts or misgivings, no debate or inquiry, whether some other *course* might not be pursued, some more plastic, less expensive *material* used. The mind must be satisfied upon this point, in a manner to leave no room to doubt before the work is commenced, or it will never be finished as it should be. The full gratification which results from the ambition and desire, to accomplish a fixed purpose and end, will never be realized, except the whole heart and soul are enlisted and engaged in the object to be accomplished.

These operations require a continued, constant, unwearied application of mind and body.

It is this which renders success so extremely difficult to be attained—this that makes it such hard labor, that few are willing to continue steadfast to the end—this reaching, anxious exercise, of reason, reflection and judgment—this intense study and application to accomplish, what the man, who has not experienced it, has no adequate conception of, and fails to comprehend—this, that sometimes occupies the mind by night as well as by day, and which the poet has so vividly portrayed, that the artist at once realizes the picture :

"The thoughts that bear me company the livelong day,
Through my thin sleep look in upon me still,
And yet methinks the darkness seems to bring
Light of its own ; for waking oftentimes,
In the dead lonely hush, the painted world
I labor in by day, starts suddenly
On the dark void, as if a master hand
Stamped it upon the curtain of the night ;
And parts that many an hour I've wearied o'er,
Often effaced, and vainly still renewed,
Arrange themselves in shapes of wondrous power ;
Then I lie tossing, wearying for the sun,
That I may haste to fix them here forever."

To continue this subject yet a little farther, it is our duty, also, to warn those who are possessed of superior talent, and are striving to climb the rough ascent to popular distinction and eminence, as well as to fortune, that it is a hard hill to climb, there are a great many slippery places, a great many opposing obstacles, and inducements to turn to the right or to the left, to look for some easier, readier way to reach the summit, instead of following the straight and narrow path, by which alone they can ever attain the realization of their hopes and desires. The majority of those who start upon their professional career, do so with the expectation, and intention of doing all that in them lies to make the most money in the shortest possible time. This is not the way to attain eminent rank and high station.

They should look to a proper performance of all their operations, to their improvement in these, and should strive to the utmost extent to multiply the more difficult evidences of their skill, and that too for many years, without regard to any immediate appreciation of their works, or adequate remuneration for their services. They cannot know themselves, what is the true value and price of their labor until time has proved it. But they will find eventually, after a lapse of many years it may be, that if they have been successful in those cases which have cost them the greatest amount of time and labor to execute, they will be repaid an hundred fold.

"The bread which has been cast upon the waters will return to them;" and "as they have sown so will they reap fame, fortune, honor, and in addition to these, the happiness of a mind at peace with itself, and the gratitude of those who will cherish their memory with fond recollection, when from age or infirmity they are no longer able to minister to the suffering, or when they shall have passed away from the scene of their labors upon earth," and the "places which have known them, shall know them no more forever." How dear to every heart, how prized above all price should be this reflection. That our memories shall be cherished here upon earth for the *good deeds* we have done, when we are no more.

I hardly know of two words in the wide range of our language which combine to express more lively emotions, more heart stirring and exalting pathos, than the simple phrase, "Remember me."

It forms a golden chain of love and memory, that connects the present to the unforgotten past, along whose shining links is conveyed, all that we have ever known or felt, or enjoyed in life's toilsome journey. Neither time with its wasting hand, nor separation from the scenes of childhood, and early manhood, and more than all from the loved ones, that were all the great world to us then, can ever efface from memory's faithful tablet, those two words so deeply engraved thereon.

All the life, and the thousand hallowed associations connected with it, are recorded in that simple phrase, "Remember me."

And do we not all feel in our inmost hearts the strong, ardent, burning desire, that our memory shall be cherished, when we are absent from loved friends, that we shall not be entirely forgotten, when we shall have taken our final departure, "to that bourne from whence no traveller returns."

Is not this thought an incentive to a life of well doing, to a self-sacrificing, self-devoting interest in the cause of humanity.

Do we not realize that the spell which hangs around those two simple words is a spell to win us from the path of error, to animate us, to impel us (even if there was no higher, holier incentive to lead a good life) to renewed exertion, so that we may leave behind us a large circle of grateful friends, whose regard, veneration, and long cherished love for our memory will be a prouder monument than sculptured tomb or marble mausoleum?

I have endeavored to impress upon you the need there is for greater zeal and activity in the pursuit of knowledge, and its promulgation.

I do not wish to detract in the least, from the merit of those who have striven hard for the last twenty or forty years to the accomplishment of this end. I do not undervalue the good which has been done.

Our colleges have accomplished much, *very much* to advance the character and respectability of the profession. We *need* public schools, colleges, and educated instructors. The superficial knowledge, often worse than none, too generally obtained by passing a few months in the office of some obscure practitioner cannot fit men for practice. We owe to those who have supplied this demand at great personal sacrifice, and often at pecuniary loss, our meed of thanks. I do not believe that in any private school in the land, and with the best practitioner in the profession, a student can be enabled to acquire the same extended information, equal advantages, or general fitness, to commence the practice of his profession that he is able to acquire in some of our colleges. And why? Because practitioners of ability and eminence, have such a requisition made upon them for their services, such a continual demand to answer the calls for their professional aid and assistance, that they cannot find the time to devote to this purpose.

The editor of one of our public journals, in animadverting upon some remarks of mine upon this subject, which were published a few years since, "*indirectly and negatively, if not directly and positively,*" lent his influence to justify charlatanism, by advancing the plea, that the charges of eminent practitioners were so exorbitantly high, that pupils had been *forced* to enter the offices of men who were not so well qualified as instructors.

I will quote his own language upon that point, to show the utter inconsistency of his argument; the radical spirit with which the leading articles of that journal have been too often imbued, *unimportant,* only that such doctrines sometimes exert a pernicious influence and tendency upon those who are entering the profession. The reasoning, reflecting mind, can see the incongruity of such ideas at once, and it needs only a common sense view of the question to be convinced how much of conformity to fact, or reality, it is deficient in. It speaks for itself; it needs no argument to prove its absurdity.

He says, "That there is much truth in these alleged causes of superficial education, and consequent bad practice among dentists, we freely admit; but we apprehend that if all the causes were examined and exposed, it would be found, that some of these very liberal minded men, whom Dr. Foster so much delights to honor, have indirectly and negatively, if not directly and positively, lent their influence to produce this very imperfection in dental education."

"Twenty-five or thirty years since, the success of a few eminent dentists in this country, drew public attention to the importance of operations upon the mouth, for the better preservation of natural teeth, as well as repairing their loss by artificial means."

"The demand was then great for skillful and scientific dentists, and yet the terms for tuition were so exorbitantly high, as to preclude the possibility of many, who would gladly have availed themselves of their instruction, from doing so. From one hundred to one thousand dollars was frequently asked for instructing students in dental surgery. The consequence was, that but few could afford to pay for their instruction, and were forced to enter the offices of men who were less qualified to instruct, or commence without any instruction at all, and trust to subsequent observation and experience to perfect them in their operations." "Young dentists," he added, "were watched with a most jealous eye. If one entered their office, he was regarded as a spy, and if he asked any questions relating to practice, he received only evasive answers." And then publishes the following note, taken, as he says, from Mr. L. S. Parmly's Lectures, published in 1820, soon after his return from Europe, and very unjustly censures him for rating his services so high. "Mr. Parmly, being desirous that his peculiar treatment of the teeth, his operations, and general views upon the subject, should be as widely diffused as possible, for the common benefit of society, undertakes to qualify gentlemen of liberal education, for practice, as dentists, on the following terms: For practice in London, $1,000. In any other city of Great Britain or America, $700. For foreign practice, $500. These terms apply solely to a finished course of instruction, including every particular of the art with which he is acquainted."

Now, I would ask any candid liberal minded man in the profession, or out of it, considering the great demand there was at that period, 1820, for well educated men in the profession, and the value of time to those who were engaged in it, in answering the demand made for their aid and assistance, and also the large field there was, in which all who were properly and thoroughly instructed in the principles of practice, might enter with the absolute certainty of reaping a rich and abundant harvest, whether $500, or $700, or $1,000, should be considered "*so exorbitantly high*?" Would not any man possessing that amount of capital, invest it at once in any other business in which he would feel as certain of realising a rich return? Again, who among you, in full practice now, has hours entirely occupied with professional labor, could afford to appropriate that portion of time, which, in justice to a pupil, he would feel obligated to do, if he performed his duty, *his whole duty*, to that pupil so as to fit him to act well his part, that could afford to do it, for a less sum, than the *highest price* which has been quoted; with the advantages offered for instruction in our colleges, I cannot see, amongst dentists *in full practice*, how any could conscientiously discharge his duties as an operator, and devote as much time to the private instruction of pupils also, as would be indispensably necessary to enable them to compete in all requisite qualifications with the graduates of our colleges, without manifest detriment to himself, in a pecuniary point of view, at anything like the ordinary prices of a collegiate course of education.

In view of the great and increasing demand there is for good and efficient operators, the profession and the community have alike reason to rejoice, that men have been found both able and willing to become public instructors, and take this heavy responsibility upon themselves.

To return from this digression, I would like to be informed, how young men were "forced," because they had not the *means to pay*, for the best instruction, were "*forced*" to go to those who were practicing without knowledge, or (utter absurdity) "*commence without any instruction at all, and trust to subsequent observation and experience to perfect them in their operations?*" Forced, forsooth! they had much better be "hewers of wood, and drawers of water," than hewers and drawers of teeth, and would then be *honored* in their occupation.

Could they not have embarked in some honest calling, and industriously have pursued it, until they had *acquired* the means of paying for a knowledge of the profession they desired to adopt?

Instances are related of dentists who have entered the offices of distinguished operators, without a proper introduction, in doing which, they were *very justly* regarded, in the light our artiste editor alludes to.

It is customary when an application is made for our services, to record the name of the applicant, and if one claiming to be a member of the profession, enters the office of an eminent dentist with a friend, who is to be the patient, and watches the manner and method of conducting an operation, or becomes himself the patient, without disclosing his name and occupation, the mildest appellation that could be applied to him, would be a "*spy.*"

*I do not believe* that a dentist of good standing, who had just claims to be considered within the pale of the profession, should enter the door of any of "those liberal minded men,"

to whom this gentleman alluded, ***declare his name and business***, as every high minded, honest man would, and ask for information, or professional aid, without its being chéerfully rendered.

Those only are likely to be watched with "*jealous eyes*," and receive "*evasiv eanswers*" whose lion skin is not sufficiently ample to cover up those long ears, by which the true asinine species is so easily recognised, and

> "Who compound for sins they are inclined to,
> By damning those they have no mind to."

My object in dwelling upon this point at such length is, to show that there are men claiming to be considered the exponents and propounders of professional rules and orders, who are disposed to cavil at the fees which eminent men in the profession are justly entitled to.

If they demand and receive such a price for their time and talents, as they value them at, from those who rightly estimate their worth, must they be *compelled* to make a great personal sacrifice, to meet the wants and circumstances of others, who, because they have not the capital to invest, would insist upon their right of possession on more liberal terms.

Is not knowledge *property*? Does not he who possesses it hold a legal title, and right of ownership, and the power of disposing of it, to make profit by it, at his own valuation? Have others the right to dictate, or to demand that it shall be transferred to them, for a minor consideration, or failing in this, to seek its attainment surreptitiously?

Has not he who has attained wisdom, paid a price for it, labored harder for it, than those who dug in the bowels of the earth for gold, or dive into the caverns of the deep after pearls? Is he who possesses the greatest treasure, the largest stock of knowledge, incapable of estimating the true worth of it? And if he does not feel inclined to dispose of it to those who feel their own deficiency and barrenness, at their low estimate of its value, are they to be justified in *stealing it*?

No one is under *obligations* to pay to the best instructors, the high prices demanded. An applicant may pay whatever he chooses elsewhere. If *he* decides, that he can obtain all the knowledge he requires for a picayune, why that will be its cost to him; let him pay it, and no grumbling. His patients will most likely, be the only fault finders.

If, on the other hand, those perfectly able to pay, who choose to invest their capital in buying professional stock, shall see fit to consider, that it does *make some difference*, where and how they invest it, and prefer to buy all the knowledge they can, and expend thousands for it, whose business is it but their own. Who should cavil if they are satisfied? Who that has paid the largest sums for instruction, would not now willingly pay ten times the amount, rather than be compelled to practice this profession, with any diminution of his intellectual, physical, or mechanical acquirements and resources? Knowledge, is our most available and reliable operative agent, the only sure guide that can conduct in the way of well-doing, that can lead and support us up the slippery ascent, which we must climb, before we can reach the goal of our earnest hopes and honorable desires.

It matters not in what busi ness or profession men may engage, they must take rank relatively, according to their knowledge, talents, and genius. They cannot continue on an equality, one will invariably precede another, according to his ability in using the gifts and advantages, which his own exertions have secured to him, in addition to those with which GOD has endowed him.

Energy is the true mark of genius. In the present day of steam accelerating advancement, and rapid movement, the lazy man, no matter how extraordinary his acquirement, must always fall behind in the race.

Emerson, in one of his lectures, traces, with the clear sweep of an artist, the vital necessity of energy and labor, to even the most gifted.

He says—"Genius unexerted is no more genius than a bushel of acorns in a forest of oaks. There may be epic in men's brains just as there are oaks in acorns, but the tree and the book must come out before we can measure them. There is a large class of grumblers and wishers who spend their time in longing to be higher than they are, while they should be employed in advancing themselves. These bitterly moralize upon the injustice of society.

Do they want a change? Let them change—who prevents them? If you are as high as your faculties will permit you to rise in the scale of society, why should you complain of men? It is God that arranged the law of precedence. Implead Him or be silent! If you have capacity for a higher station, take it—what hinders you? How many men would love to go to sleep, and wake up Rothschilds or Astors?

How many men would fain go to bed dunces, to be waked up Solomons. A man of mere "capacity undeveloped," is only an organized day-dream with a skim on it. A flint and a genius that will not strike fire, are no better than wet junk wood. We have scripture for it, that a "living dog is better than a dead lion." If you would go up, *go*—if you would be seen, *shine.*

At the present day, eminent position in any profession is the result of hard, unwearied labor. Men can no longer fly at one dash into eminent position. They have got to hammer it out by steady and rugged blows. The world is no longer clay, but rather iron, in the hands of its workers.

We do most earnestly assert the rights of genius and talent to such remuneration as their services will fairly command.

Arkwright's, Watt's, Whitney's, Fulton's, and a great many other beneficent inventor's achievements, either enriched them, or should have done so.

Every one is entitled to receive whatever he or she may fairly earn. If the lovers of music choose to pay the greatest living singer, one thousand or even ten thousand dollars per night; who shall say, that sum is not fairly hers? Just as clearly as any one of our eminent lecturers, is entitled to the fifty or one hundred dollars, which the people of some village or city choose to pay him for his services.

Let not those whose living comes by their own exertions disparage the claims of eminence, *fairly earned,* to its full and ungrudged recompense.

I wish briefly to allude to one other topic, which is of sufficient importance to receive more attention; but, that I have already extended this address to an undue length. It is the system of advertising. There are three modes, all equally demeaning and undignified in a professional man, though, probably, not altogether unprofitable, since they are still resorted to in various sections of the country, though not to so great an extent as in former days.

Self-laudatory advertisements, with a long array of names and recommendations published in the daily papers, is the most general mode. Next, the publication of small journals and tracts, or treatises on subjects pertaining to the teeth issued by a private individual, and distributed at every body's door, like a whitewasher's or carpet shaker's card for the public benefit. If these could be enlightened, and have the prayer answered, "O that the laird the giftie gi'e us, to see ourselv's as ithers see us," they would realise the ridicule which attaches to them, as well as the mortification which we feel, when our patients come to us, and say: Here, doctor, we have received another begging invitation from one of *your professional brethren*, and were *reminded* of you again. Lastly, the publication of indecent dissertations, and disgusting details in some of our literary weekly journals, upon matters, which every patient who employs an efficient and skilful dentist is informed of, when his or her *necessities* require it, and which should never appear in print, except in journals, especially devoted to such purposes.

I beseech you my friends to endeavor to impress upon all, who are within your reach, over whom you can exert any degree of influence—and who are worth the effort, the folly of advertising in any of these forms.

It will not increase the business of good operators to resort to these means of obtaining practice. It cannot take away from the business of other good operators, if some do resort to it.

The only class who may reap a scanty pittance thereby, are the uneducated, the unfitted, whose only hope lies in securing an unweary straggler now and then, whose eye is caught by that ever glittering, but brazen sentence : "The best operations performed at the following reduced prices, and *warranted.*"

Let this aphorism impress all who have any feelings of professional pride, and who are inclined to sacrifice them on the altar of their necessity, vanity or ambition.

"Be particularly careful not to speak of yourself when you can help it. The less you say of yourself, the more the world will give you credit for."

Whatever perfections you may have, be assured people will find them out; but whether they do or not, nobody will take them upon your word.

True genius is ever modest.

The masses of our people have a mind of their own, which is not so easily perverted as empirics, and mere panderers after popularity think it; an intelligence sufficient in its practical application, to guard them against the mischievous expedients of charlatans and demagogues, who vainly seek to practice upon their sympathy and credulity, to advance selfish ends.

The advertising fever which has been stirred up in this country by the Crawcours, Malans, and some of their followers, has abated somewhat of its malignant character, from which we are prone to deduce some wholesome conclusions, and we shall see more and more clearly, how ephemeral its effect is. It is as idle as the wind. A mere ripple on the surface making no lasting impression—a mark in the sands on the great sea shore of public opinion, which the waves of reason and reflection will soon have swept away.

My purpose has been to endeavor to impress you with a due sense of the importance of a life of industry.

It is the alpha and omega of this exhortation, and, in concluding, I would again urge you to more strenuous, self-sacrificing, self-devoting labor and zeal in your profession.

Honest industry will secure the esteem of the wise and the good, will yield the rich fruit of an easy conscience, and ensure that hearty self-respect which is above all price. Counsel all young men that they may be incited by words to good deeds.

To be diligent in business—to cultivate and improve the heart and the mind, that they may command the confidence and respect of those, whose respect is worth an effort to obtain.

Success may be slow at first, the more slow from a firm adherance to the principles of truth and justice, but none the less sure.

There will be many struggles, discouragements, disappointments, temptations, and trials of faith, constancy, honesty, firmness of purpose, stability of character, powers of endurance, which all, in the commencement of their career, have had some experience of.

There are times when heaviness comes over the heart, and we feel as if there was no hope, no inducement to exertion. Who has not felt it? For this there is no cure but *work.* Plunge into it—put all your energies in motion, rouse up the inner man—*act*—and this heaviness shall disappear as mist before the morning sun.

There arise doubts in the human mind that wrap us in gloom, and make us think it were bootless to attempt anything. Who has not experienced them? Work—that is the cure. Task your intellect—stir up your feelings—rouse the soul—and these doubts, hanging like a heavy cloud upon the mountain, will scatter and disappear, and leave you in sunshine and open day.

There comes suspicion to the best of men, and fears about the holiest efforts and we stand like one chained.

Who has not felt it? *Work.* Therein is freedom. By night, by day, in season and out of season, work, and liberty will be yours. Put in requisition mind and body, war with inertness, snap the chain-link of selfishness, stand up as a defender of the right; be yourself, and this suspicion, and these fears, will be lulled, and like the ocean storm, you will be purified by the contest, and able to bear and breast any burden of human ill.

Gladden life with its sunniest features, and gloss it over with its richest hues, and it becomes a poor and painted thing, if there be in it no toil, no hearty hard work. The laborer sighs for repose. Where is it? What is it? Friend, whoever thou art, know it is to be found alone in work. No good, no greatness, no progress is gained without it; work, then, and faint not, for therein is the well-spring of human hope and human happiness.

NOTE.—Since this address was delivered, a controversy has been started upon the subject of establishing Dental Chairs in Medical Colleges. The advocates of this experiment contend that superior advantages will be thus offered for the education of dental practitioners. Some have gone so far as to express the belief, that there is an absolute necessity for it. They contend that the course of instruction pursued in dental colleges is deficient in many important particulars, that they do not combine all the essential elements, so connected as to create a fundamental basis, that can serve as the ground work of a system of correct practice. There can be no doubt, that the appointment of a dental professor in every medical school in the country would exert a salutary influence in awakening public attention, by and through the aid of the great body of medical men, whose interest would be secured to the intimate relation which this specialty holds and exerts upon all the other departments of medical science. It would also have the effect to incite our dental colleges, to endeavor to supply deficiencies in their organization, if they are imperfect, and thus secure a more complete and effectual system.

But that a medical school, with one dental chair only, even if that chair be occupied by the best man who could be appointed to fill it, can ever be successful in making efficient *dental operators,* is an imaginary hypothesis. Other advantages must be combined therewith, to render such a scheme either feasible or practicable. And what! Thorough practical instruction in some private office, by a *competent* teacher, not for two or three *months,* but for as many years. The question then arises, will the most eminent men in the profession, with a self-sacrificing spirit, from motives of disinterested benevolence, and pure philanthropy, *give* their time and attention to as many pupils, as each would have apportioned to him, if the call of good operators, which the great increase of dental disease in this country demands, is to be answered in this way? I think not. It would be almost equivalent to a *free gift,* if they should consent to receive students, on as liberal terms as those which dental colleges offer. So long as dental colleges continue to exist, and to hold out the same inducements which they now do, will applicants consent to pay eminent private teachers, the *value* of their time and services, besides being subjected to the increased additional expense for attending full courses of lectures in a medical college? I would not be understood as intending to decry, or undervalue the utility of a thorough and complete knowledge of medicine and surgery in all its branches, to him who devotes himself to our particular specialty, the *advantages* are indisputable.

But as we cannot induce candidates to adopt our mode of thinking, and there are no laws to restrict them; let us do the best we can for them. Dr. Westcott's very able article recently published in the New York Medical Review, presents very strong and forcible reasons, why dental colleges should receive the continued countenance and support of the profession.

If they are not in all respects what they should be, they may be improved.

Let their requisitions be more imperative.

Let them increase the needed and essential qualifications for admission as well as for graduation. Let them extend the required term of study. Let them enforce all their laws and regulations, rules and orders, strictly to the letter.

Their doors have been freely opened to the profession, and their claims to respect and encouragement if freely canvassed, should be as fairly and considerately judged. Suppose there are, as there may be, each year, some who seemingly have their degrees conferred upon them, before they are qualified to be admitted to practice, in the strict acceptation of the term. May not the same be said of the graduating class of every medical school in the land? Of every classical school? Of every college? Are *private schools*, with the *best instructors*, an exception? Are their pupils, after a three years term, supposing them all to have pursued industriously and laboriously the duties required of them, are they all *equally* competent *to fill high places*? And is it right, is it just, is it reasonable to denounce and condemn dental colleges because *they* are not immaculate?

What state of mind, or of the affections is there, whether desired or deprecated, that may not minister to our annoyance, if that pure principle which brings satisfaction and strength, and harmony, to the soul, be wanting?

Mediocrity of talent, failure in a profession, is commonly considered as an occasion for intolerable disquietude; but inferiority itself is not more agitating than the situation of a proud man, exalted in the public opinion and obliged to satisfy the demands made upon an idolized reputation.

"What shadows we are, and what shadows we pursue." Ambition, sees more than it can gain; discouragement sees nothing that it can gain.

